# Skin Biopsy Targeting Senile Hemangiomas for the Diagnosis of Intravascular Large B-cell Lymphoma

**DOI:** 10.7759/cureus.75658

**Published:** 2024-12-13

**Authors:** Yuta Yoshino, Shiori Shigeta

**Affiliations:** 1 Internal Medicine, Saitama Citizens Medical Center, Saitama, JPN

**Keywords:** abnormal weight loss, fever of unknown origin (fuo), intravascular large b cell lymphoma, senile hemangioma, skin biopsy punch

## Abstract

Intravascular large B-cell lymphoma (IVLBCL) is characterized by clinical presentations described as B symptoms, consisting of fever, night sweats, and weight loss. Intravascular lymphomas are more frequently diagnosed in elderly patients and are challenging to diagnose because of their nonspecific clinical presentation. Malignant lymphomas are recognized as the leading cause of fever of unknown origin. Although random skin biopsy is useful for diagnosing intravascular lymphomas, no standardized method for random skin biopsy has been established. Methods to avoid unnecessary skin biopsies and improve diagnostic accuracy have been investigated previously. Here, we report a case of a man in his seventies with intravascular large B-cell lymphoma diagnosed by skin biopsy targeting senile hemangiomas. The combination of random skin and targeted biopsies of skin lesions such as senile hemangiomas may help avoid the diagnostic invasiveness caused by unnecessary biopsies and improve the diagnostic accuracy for intravascular lymphomas.

## Introduction

Intravascular large B-cell lymphoma (IVLBCL) is a subtype of extranodal non-Hodgkin lymphoma characterized by lymphoma cells that grow within small blood vessels and do not form an obvious extravascular mass [1]. It commonly develops in patients in their 60s and 70s. It is difficult to diagnose because of its nonspecific clinical presentation, such as fever, weight loss, and general malaise [2]. Malignant lymphoma is one of the most common causes of fever of unknown origin (FUO), accounting for 6% of all cases of FUO [3]. FUO is defined as a fever of 38.3°C or higher that persists for at least 3 weeks and is unexplained after 3 days of hospitalization [4]. FUO is further divided into four categories: classical FUO, healthcare-associated FUO, immune-deficient FUO, and HIV-related FUO.

IVLBCL is classified into the classical and Asian variants based on regional differences in symptom manifestations. The Asian variant is more frequently associated with hemophagocytic syndrome and hepatosplenomegaly, although skin lesions develop less frequently than in the classical variant [5]. Here, we report a case of IVLBCL in which intravascular lymphoma was suspected based on clinical features and diagnosed at an early phase by skin biopsy targeting senile hemangiomas.

## Case presentation

A 73-year-old man with a history of hypertension presented to our hospital with a complaint of appetite loss for a month. The patient had been receiving oral nutritional supplements for three weeks because his food intake had decreased, and he experienced a weight loss of 10 kg in a month. There were no gastrointestinal symptoms, such as vomiting or diarrhea, and tar stools were not observed. Although the patient did not have night sweats, he continued to have a fever (maximum body temperature: 37.5°C). His blood pressure gradually decreased, and his regular antihypertensive medication was withdrawn. His vital signs were as follows: temperature, 37.4°C; blood pressure, 132/88 mmHg; heart rate, 117/min; and SpO_2_, 98% in room air. Physical examination did not reveal anemia of the conjunctiva or ocular jaundice. His respiratory sounds were normal, and no compensatory breathing was observed. There were no cardiac murmurs or leg edema. Blood tests at the first visit to our hospital revealed hyponatremia (132 mEq/L) and the following findings: serum albumin, 2.3 g/dL; creatinine, 1.07 mg/dL; lactate dehydrogenase (LDH), 1256 U/L; soluble interleukin-2 receptor (sIL-2R), 4375 U/mL; and C-reactive protein, 14.57 mg/dL (Table 1).

**Table 1:** Haematological and biochemical test at the first visit. MPO-ANCA: myeloperoxidase-antineutrophil cytoplasmic antibody; PR3-ANCA: proteinase3-antineutrophil cytoplasmic antibody.

Variable	Result	Normal values
White cell count (/mm^3^)	7500	3500-9700
Hemoglobin (g/dL)	10.2	13.6-18.3
Platelets (*10^4^/µL)	19.4	14.0-37.9
Albumin (g/dL)	2.3	3.7-5.5
Total bilirubin (mg/dL)	0.7	0.3-1.2
Lactate dehydrogenase (U/L)	1256	120-245
Urea nitrogen (mg/dL)	28.0	8-20
Creatinine (mg/dL)	1.07	0.65-1.09
Sodium (mEq/L)	132	135-145
Potassium (mEq/L)	4.6	3.5-5.0
Chloride (mEq/L)	100	98-108
Calcium (mg/dL)	8.0	8.6-10.2
Serum iron (µg/dL)	12	60-210
Total iron binding capacity (µg/dL)	131	250-410
Ferritin (ng/mL)	1714.3	21-282
Vitamin B12 (pg/mL)	305	233-914
Folic acid (ng/mL)	6.1	3.6-12.9
Thyroid-stimulating hormone (µIU/mL)	1.12	0.35-4.94
Free-T4 (ng/dL)	0.90	0.70-1.48
Adrenocorticotropic hormone (pg/mL)	5.9	7.2-62.3
Cortisol (µg/dL)	21.4	4.5-21.1
C-reactive protein (mg/dL)	14.57	0.0-0.3
Soluble interleukin-2 receptor (U/mL)	4375	122-496
MPO-ANCA (IU/mL)	0.2	0.0-3.4
PR3-ANCA (IU/mL)	<0.6	0.0-1.9
Immunoglobulin G (mg/dL)	987	820-1740
Immunoglobulin A (mg/dL)	219	90-400
Immunoglobulin M (mg/dL)	36	31-200

**Figure 1:**
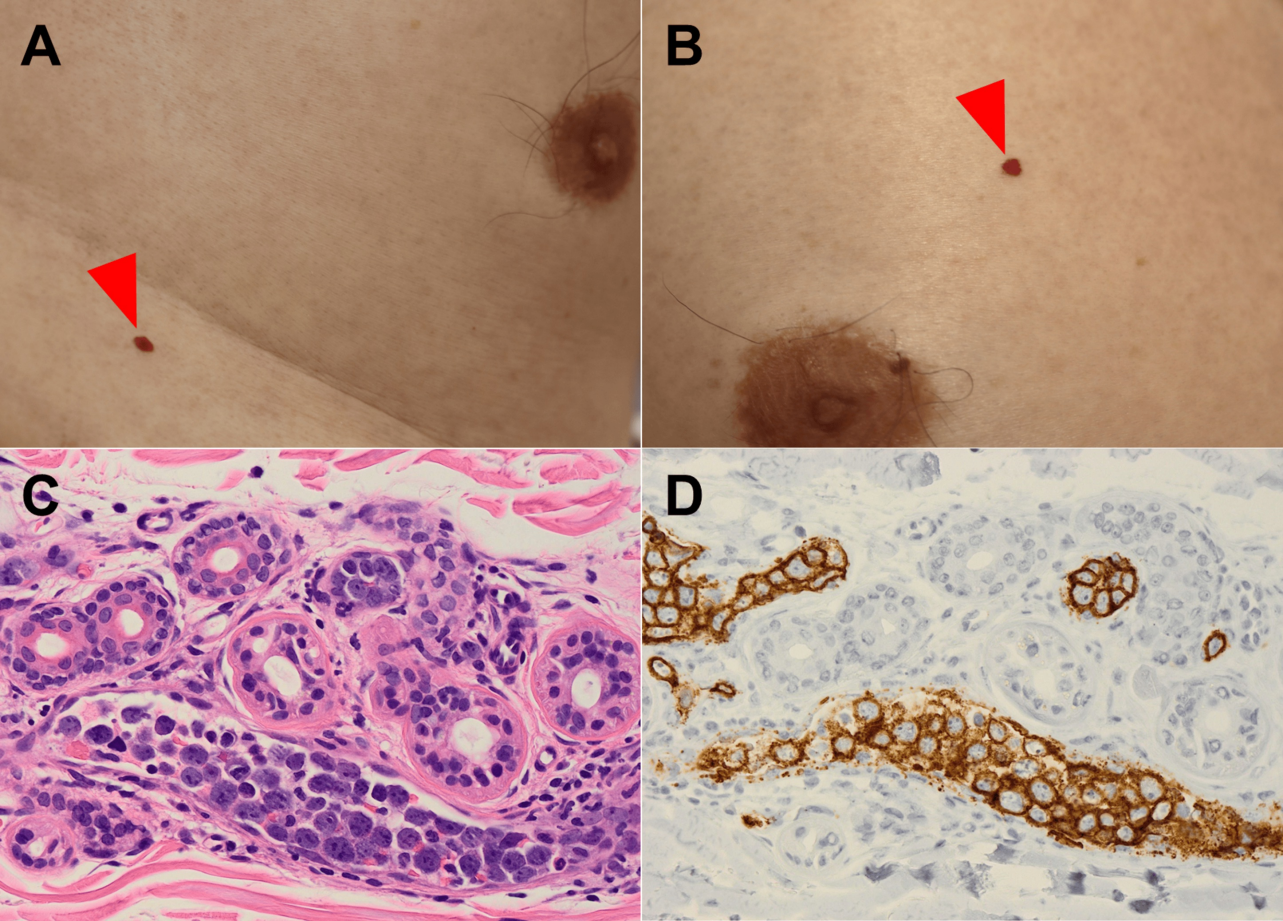
Skin biopsies and histological findings. Skin biopsies targeting senile hemangiomas. (A, B) Skin biopsy specimens obtained from the thoracic region (arrows). (C) Skin biopsy confirmed atypical lymphocytes (hematoxylin and eosin staining; magnification: ×400). (D) Skin biopsy confirmed CD20-positive lymphocytes (immunohistochemiacal staining; magnification: ×400).

In addition, no endocrinological abnormalities, such as hypothyroidism or adrenal insufficiency, were found, and the patient tested negative for serum antinuclear and antineutrophil cytoplasmic antibodies. Blood cultures were negative, and no viral infections, such as that with HIV, were observed. A plain computed tomography scan of the whole body revealed no lesions suggestive of malignancy and no enlarged lymph nodes. Because no abnormalities that could have caused anorexia were observed on esophagogastroduodenoscopy, intravascular lymphoma was considered in the differential diagnosis. Skin biopsies were performed by a dermatologist; the biopsies targeted two senile hemangiomas in the thoracic region (Figure 1A, Figure 1B). Atypical lymphocytes were confirmed inside the hemangioma and small vessels of the dermis (Figure 1C). CD20 immunohistochemical staining was positive for atypical lymphocytes within the small vessels, confirming the diagnosis of IVLBCL (Figure 1D).

## Discussion

Patients with IVLBCL present with B symptoms, including fever, night sweats, and weight loss. Because the symptoms are nonspecific, the diagnosis of intravascular lymphoma is difficult [2]. IVLBCL is among the differential diagnoses of FUO, and the average duration of FUO is two to six months [6]. Bone marrow or random skin biopsy is useful in the diagnosis of intravascular lymphoma because both are minimally invasive and simple histological diagnostic procedures.

However, there are no standardized biopsy methods for random skin biopsies in the literature, and there is no formalized algorithm yet to assist in the diagnosis of IVLBCL [7]. To avoid unnecessary skin biopsies and improve diagnostic accuracy, MacGillivary et al. recommended three to four skin biopsies from the thigh, abdomen, and/or posterior upper arm, performed via either an incision or telescopic punch biopsy [8]. The benefit of senile hemangioma biopsy for the diagnosis of intravascular lymphoma has been previously reported, and senile hemangiomas should be biopsied, if present [9]. Random skin biopsies have been reported to be useful for diagnosing IVLBCL, even in the Asian variant, where skin lesions are less common than in classical variants [1].

In the present case, the patient presented with nonspecific systemic symptoms such as anorexia and weight loss, and the presence of malignant lymphoma was suspected based on significantly elevated LDH and high sIL-2R levels. Because no enlarged lymph nodes were found, a skin biopsy was performed to diagnose intravascular lymphoma. As a requirement for a random skin biopsy to be useful in the diagnosis of intravascular lymphoma, one or more of the following four findings consistent with FUO without lymphadenopathy or hepatosplenomegaly are necessary: hematological abnormalities, elevated serum LDH levels, hypoxemia, and abnormal neurological symptoms [10]. Although no definitive diagnostic criteria for random skin biopsies have been established, a newly developed IVLBCL probability scoring system has successfully stratified groups with high diagnostic sensitivity for IVLBCL [11]. The advantage of combining random skin biopsy with targeted biopsy of skin lesions such as senile hemangiomas has also been reported in the histological diagnosis of intravascular lymphoma [12]. Random skin biopsies improve the diagnostic accuracy of IVLBCL as the number of samples increases [13]. However, an appropriate number of biopsies should be considered since a higher number of biopsies would unnecessarily increase the amount of invasiveness received by the test.

## Conclusions

Because the symptoms of intravascular lymphoma are nonspecific, diagnosis is difficult. The presence of intravascular lymphoma must be recognized, and diagnostic techniques such as random skin biopsy should be used more efficiently. In the present case, skin biopsy targeting senile hemangioma contributed to the diagnosis of intravascular lymphoma. The combination of random skin and targeted biopsies of skin lesions may help to avoid the diagnostic invasiveness caused by unnecessary biopsies and improve the diagnostic accuracy for intravascular lymphomas.
